# Association between atopic dermatitis and risk of stroke: a systematic review and meta-analysis

**DOI:** 10.3389/fneur.2025.1630671

**Published:** 2025-07-18

**Authors:** Hong Pan, Qing-Ping Han, Min-Ling Zeng, Fang Wang, Ying Xiong, Bo Wu, Hai-Ying Yu

**Affiliations:** ^1^Department of Internal Medicine, Shenzhen Maternity and Child Healthcare Hospital, Women and Children’s Medical Center, Southern Medical University, Shenzhen, Guangdong, China; ^2^Department of Dermatology, Shenzhen Maternity and Child Healthcare Hospital, Women and Children’s Medical Center, Southern Medical University, Shenzhen, Guangdong, China; ^3^Department of Neurology, The Third Clinical Medical College of China Three Gorges University, Gezhouba Central Hospital of Sinopharm, Yichang, China

**Keywords:** atopic dermatitis, stroke, meta-analysis, risk, systematic review

## Abstract

**Background/objectives:**

Recent studies have sought to determine the correlation between atopic dermatitis (AD) and the occurrence of stroke; however, these investigations have not reached a consensus. Consequently, our team conducted this meta-analysis and systematic review to further explore the potential relationship between these two conditions.

**Methods:**

A comprehensive literature search was conducted across PubMed, Embase, and Web of Science from their inception until January 2025 to identify observational studies examining the association between atopic dermatitis and stroke risk. Random-effects meta-analyses were performed using the generic inverse variance method, and hazard ratios (HRs) with 95% confidence intervals (CIs) were calculated. Additionally, sensitivity analyses and subgroup analyses were conducted to assess the stability of the results and explore potential sources of heterogeneity.

**Results:**

This meta-analysis included 12 observational studies, comprising 8 cohort studies, 2 case–control studies, and 3 cross-sectional studies, with a total of 14,517,146 participants. The analysis revealed a significant correlation between AD and the risk of stroke (*n* = 17, OR = 1.27, 95%CI = 1.14–1.43). Subgroup analyses indicated a particularly strong association among males (*n* = 3, OR = 1.31, 95% CI = 1.07–1.60) and in the context of ischemic stroke types (*n* = 5, OR = 1.14 95%CI = 1.00–1.30). Furthermore, sensitivity analyses demonstrated that the results were stable and reliable.

**Conclusion:**

AD is independently associated with an increased risk of stroke, especially in men, and there is a notable association with ischemic stroke. Moreover, the risk appears to be positively correlated with the severity of AD.

**Systematic review registration:**

Identifier INPLASY202550006.

## 1 Introduction

Atopic dermatitis (AD) is a common, chronic, and recurrent inflammatory skin disorder primarily characterized by pruritus, which often intensifies at night. Scratching can exacerbate skin lesions, leading to further complications. Patients with severe cases require multidisciplinary management involving the dermatology, allergy, and psychology departments. Currently, the incidence of AD in children ranges from 15 to 20%, while in adults, it is between 1 and 3% in developed countries (1, 2). Notably, there has been a significant annual increase in new cases among adults in recent years (3). Ongoing research on AD has highlighted its substantial association with various allergic diseases, autoimmune disorders, infectious diseases, metabolic and cardiovascular conditions, and psychosocial issues (4–7).

Cerebrovascular diseases primarily encompass ischemic and hemorrhagic strokes. According to data from the Global Burden of Disease Study (GBD), stroke was the second-leading cause of death and the third-leading cause of death and disability combined in 2019 (8). Approximately 12.2 million new stroke cases occur annually, contributing to a global mortality rate of around 6.55 million deaths per year (8). About 44 million stroke survivors experience significant functional impairments, while an estimated 5 million individuals endure permanent severe disabilities (9). Notably, China bears the highest burden of stroke globally, with approximately 3.94 million new cases annually, resulting in about 2.19 million deaths due to stroke and 45.9 million Disability-Adjusted Life Years (DALYs) (10, 11). According to data from the World Stroke Organization (WSO) and the Global Burden of Disease (GBD), the estimated annual treatment costs for stroke worldwide exceed 721 billion US dollars, with indirect costs approximated at around 450 billion US dollars (8). Researchers have conducted extensive investigations into the pathogenesis of cerebrovascular diseases. In addition to the common risk factors associated with this condition, the relationship between AD and stroke has garnered increasing attention in recent years; however, a consensus has yet to be reached. Some studies have identified a significant correlation between AD and stroke, particularly ischemic stroke (6, 12, 13). A meta-analysis published in 2018 supports this perspective (14), while other studies have not corroborated these findings (15–17).

Due to the lack of a unified conclusion in existing studies, our team conducted a meta-analysis that integrates previous research with the most recent published findings. The aim of this analysis is to further explore the correlation between AD and stroke, thereby providing a scientific basis for the development of effective stroke prevention strategies.

## 2 Methods

### 2.1 Protocol and registration

We conducted this systematic review in accordance with the Preferred Reporting Items for Systematic Reviews and Meta-Analysis (PRISMA) statement (18). The protocol for this systematic review was registered on INPLASY (NO. 202550006) and is available in full on inplasy.com (https://doi.org/10.37766/inplasy2025.5.0006).

### 2.2 Search strategy

PubMed, Embase, and Web of Science were searched from the inception of these databases through January 2025 to identify observational studies that examined the association between atopic dermatitis and the risk of stroke. The reference lists of relevant articles were examined to supplement the search. The search strategy was devised using a combination of Medical Subject Heading (MeSH) terms, and free text searching of the title and abstract, without language restrictions. The detailed search strategies used were as follows: (atopic dermatitis OR atopic eczema OR dermatitis OR eczema) AND (stroke OR cerebral infarction OR brain infarction OR cerebral hemorrhage OR intracerebral hemorrhage OR transient ischemic attack OR cerebrovascular disorders OR cerebrovascular disorders OR cerebrovascular accident). The full search strategy for PubMed,as an example, can be found in the online Supplementary Table S1.

### 2.3 Study selection

In our meta-analysis, studies were selected based on specific inclusion criteria: (1) the study design was restricted to cross-sectional, cohort, or case–control studies; (2) effect measures included either unadjusted or adjusted odds ratios (OR), risk ratios (RR), and hazard ratios (HR), along with their corresponding 95% confidence intervals (CI). When specific data were unavailable, they were calculated from raw data whenever possible; (3) sample sizes were defined within a specified time frame. The exclusion criteria were as follows: (1) abstracts, case reports, editorials, guidelines, protocols, book chapters, and letters; (2) studies that did not employ analytical or descriptive designs with control groups; and (3) studies that lacked any database results. Title and abstract screening were independently conducted by two reviewers to determine potential eligibility, and full-text articles were subsequently assessed for final eligibility. Any discrepancies regarding selection were resolved through consultation with a third reviewer.

### 2.4 Data extraction

The following variables were extracted from each study: the first author, the country of study, the type of AD and stroke, the study design, the publication year, the subjects involved, the sample size, the percentage of male participants, the duration of follow-up or study period, the mean age or age group, as well as the odds ratios (OR), risk ratios (RR), and hazard ratios (HR), both adjusted and unadjusted, along with their respective 95% confidence intervals (CIs) and adjusted confounding variables. Two reviewers independently compared the selected data and resolved any discrepancies through consultation to ensure the accuracy and reliability of the analysis.

### 2.5 Assessment of quality

Two reviewers independently assessed the methodological quality of case–control and cohort studies using the Newcastle–Ottawa Scale (NOS) (19). The NOS assigns a maximum of 4 points for selection, 2 points for comparability, and 3 points for exposure or outcome. The studies were classified as low, moderate, or high quality based on NOS scores of 1–3, 4–6, and 7–9, respectively. The Agency for Healthcare Research and Quality (AHRQ) (20) checklist was employed to evaluate the quality of cross-sectional studies, with scores ranging from 0 to 11. AHRQ scores of 4–7 and 8–11 indicated moderate and high quality, respectively (21).

### 2.6 Statistical analysis

Meta-analyses were conducted using Review Manager software (Version 5.3) to calculate odds ratios (ORs) and 95% confidence intervals (CIs) for evaluating the association between atopic dermatitis and stroke. A random-effects, generic inverse variance method, as proposed by DerSimonian and Laird, was employed to estimate the pooled OR and 95% CI. Given that the outcome of interest was relatively uncommon, we treated relative risk (RR) or hazard ratio (HR) as equivalent to OR (22). In instances where both unadjusted and adjusted OR/HR/RR were reported, the adjusted values were prioritized. The Cochrane Q-test was performed to assess the heterogeneity among studies. A *p*-value of less than 0.10 for the Q-test was deemed statistically significant. Furthermore, the I-squared statistical test was used to evaluate the extent of heterogeneity: an I^2^ value of 0% indicated no heterogeneity, 25–50% indicated low heterogeneity, 50–75% indicated moderate heterogeneity, and values exceeding 75% indicated high heterogeneity (23). Statistical significance was defined as a *p*-value < 0.05. Subgroup analyses were performed based on study design, gender, region, and type of stroke. Furthermore, sensitivity analyses were conducted by sequentially removing each study to evaluate the stability of the results. Additionally, the funnel plot was used to evaluate the potential for publication bias using Review Manager software (Version 5.3).

## 3 Results

### 3.1 Selection

**Figure 1:**
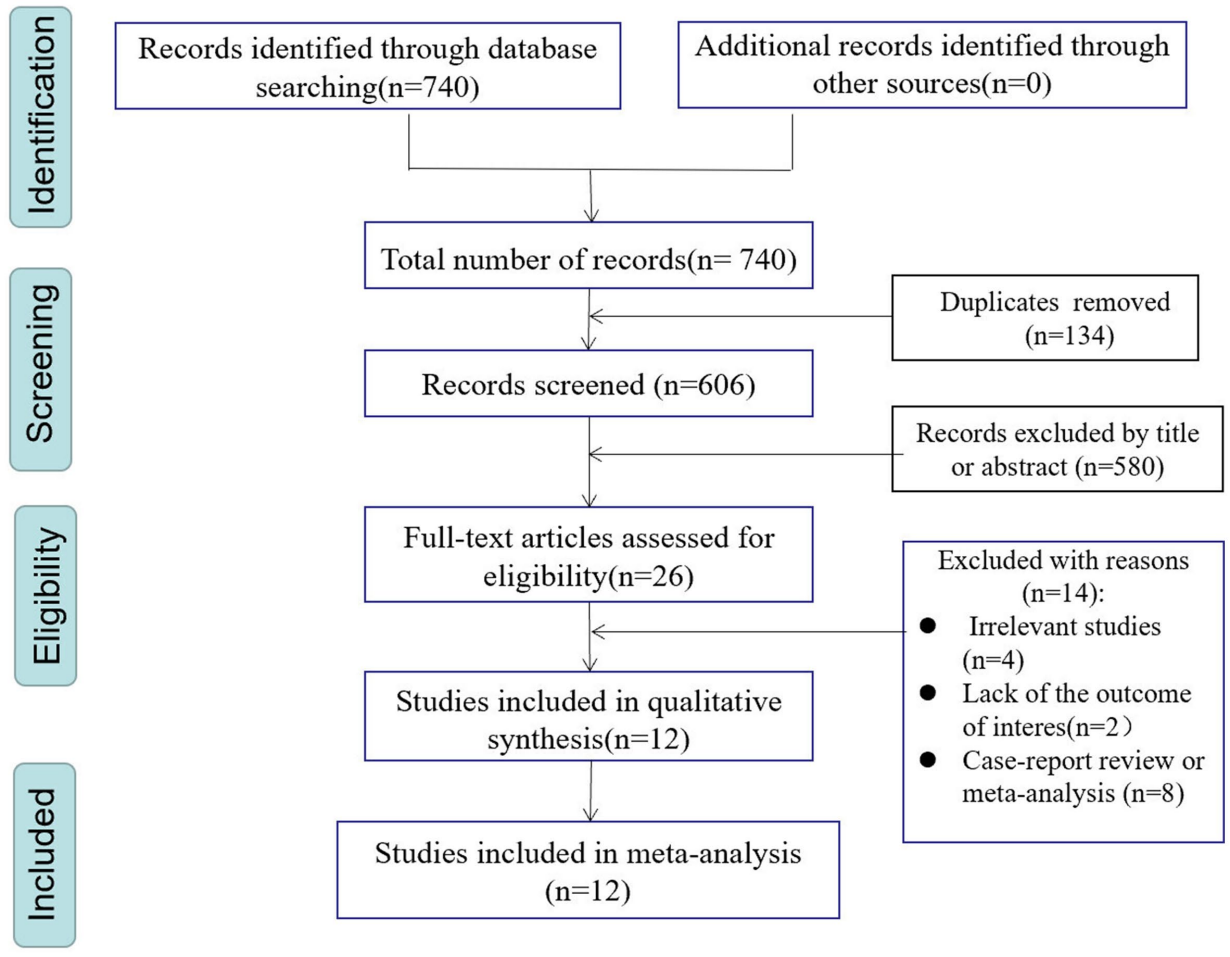
PRISMA flowchart of study selection process.

The search strategy identified 740 potentially relevant articles from PubMed (*n* = 63), Embase (*n* = 431), and Web of Science (*n* = 246). A total of 134 records were excluded due to duplication. 580 records were excluded after carefully scanning titles and abstracts. After conducting title and abstract screenings, 26 studies were selected for full-text reading, of which 12 (6, 12, 13, 15–17, 24–29) met the eligibility criteria (details of excluded articles can be found in Supplementary Table S2). Figure 1 describes the process of literature screening.

### 3.2 Study characteristics

The main characteristics of the included studies are summarized in Table 1. This review encompasses a total of eight cohorts (12, 15, 17, 24, 26–29), two case–control studies (6, 16), and three cross-sectional studies (13, 25, 26) published between 2014 and 2023. These studies were conducted across three continents: four in Asia (16, 17, 27, 28), five in Europe (6, 15, 24, 26, 29), and three in North America (12, 13, 25). The sample sizes of the included studies varied significantly, ranging from 40,646 to 5,522,341 participants. Regarding the classification of AD, seven articles (6, 15–17, 24, 27, 29) were categorized into severity levels, including mild, moderate, and severe, whereas five articles (12, 13, 25, 26, 28) remained unclassified. Regarding the types of stroke, two articles (17, 28) encompassed both ischemic and hemorrhagic strokes, three articles (6, 24, 27) concentrated exclusively on ischemic stroke, while seven articles (12, 13, 15, 16, 25, 26, 29) did not specify the type of stroke. The quality of ten studies (6, 12, 15–17, 24, 26–29) was assessed using the Newcastle-Ottawa Scale (NOS), which yielded scores ranging from 6 to 7. Furthermore, three studies (13, 25, 26) were evaluated based on the Agency for Healthcare Research and Quality (AHRQ) criteria, resulting in scores between 5 and 8 (see Supplementary Table S4).

**Table 1:** Main characteristics of included studies.

First author (year)	District	Study design	Follow-up duration (year)	Type of AD	Type of stroke	Sample size	Mean age or age group (years), male (%)
Andersen (2016)	Denmark	Cohort study	14	Mild severe	Ischemic stroke	174,797	24.5 (46.11%)
Drucker (2016)	US	Cohort study	1	NR	NR	78,702	NR (0%)
Drucker (2017)	Canadian	Cross-sectional study	NR	NR	NR	259,119	30–74 (38.6%)
Jung (2021)	Korea	Case–control study	NR	Clear mild moderate severe	NR	2,780,356	>15 (49.6%)
Lee (2023)	Korea	Cohort study	5.1 ± 2.9	Non-severe severe	Ischemic stroke Hemorrhagic stroke	81,024	50 (40.62%)
Lina (2019)	Swedish	Case–control study	NR	Non-severe severe	Ischemic stroke	1,127,267	>15 (33.87%)
Silverberg (2015)	United States	Cross-sectional study	1	NR	NR	66,652	NR
Silverwood (2018)	UK	Cohort study	5.1	Mild moderate severe	NR	1,915,916	43 (34%)
Stand (2017) (Co)	Augsburg	Cohort study	7	NR	NR	1,214,133	64 (43.10%)
Standl (2017) (Cr)	Augsburg	Cross-sectional study	3	NR	NR	1,180,678	65 (44.33%)
Su (2014)	Taiwan	Cohort study	5	Mild moderate severe	Ischemic stroke	40,646	40 (38.1%)
Sung (2017)	Taiwan	Cohort study	8	NR	Ischemic stroke Hemorrhagic stroke	75,515	33.5 (45.8%)
Wan (2023)	UK	Cohort study	5	Mild moderate severe	NR	5,522,341	Pediatric 4 (51.75%) Adult 47 (45.7%)
NR: No Report.							

### 3.3 Association between AD and risk of stroke

**Figure 2:**
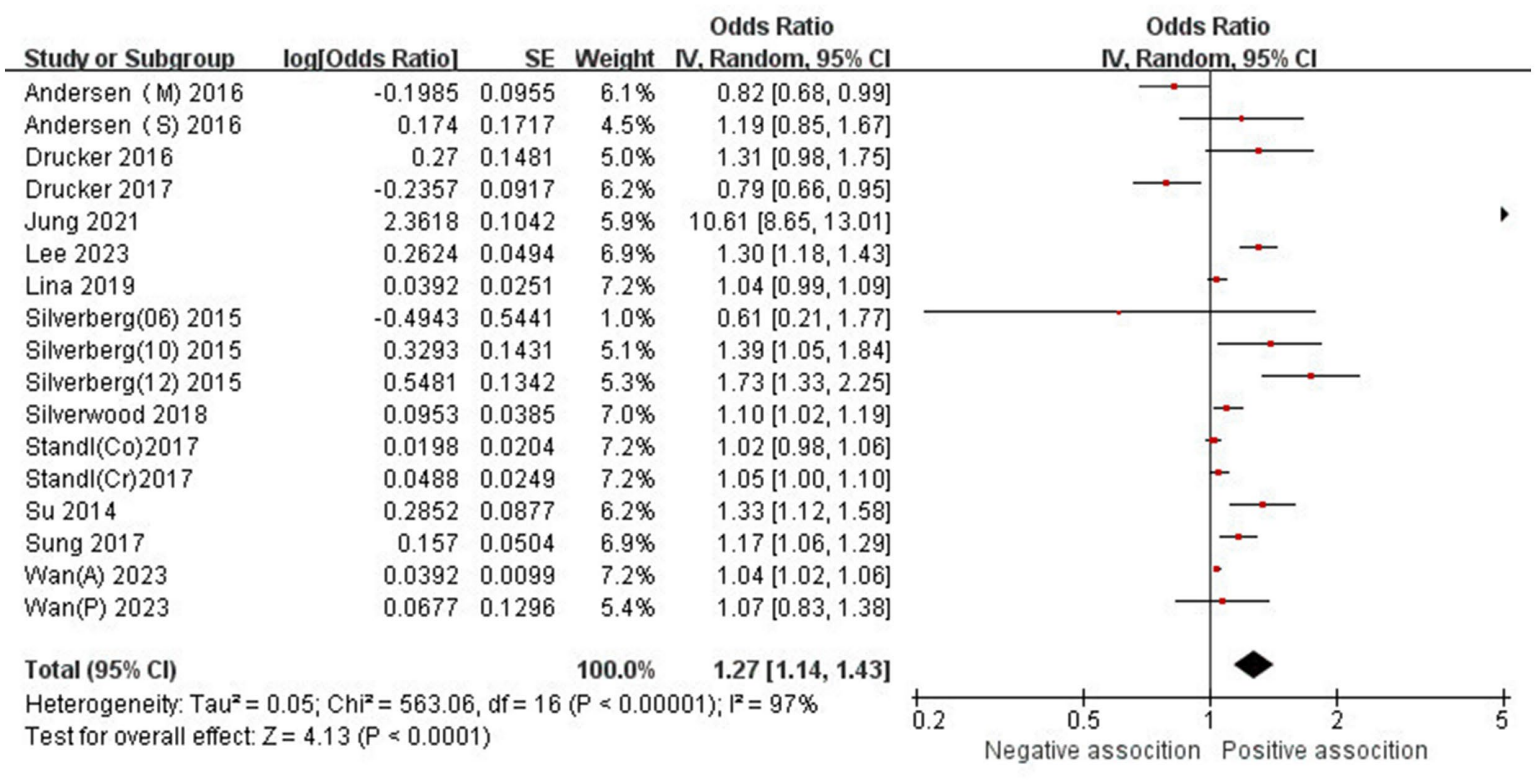
Forest plot of evaluating the association between AD and stroke.

**Figure 3:**
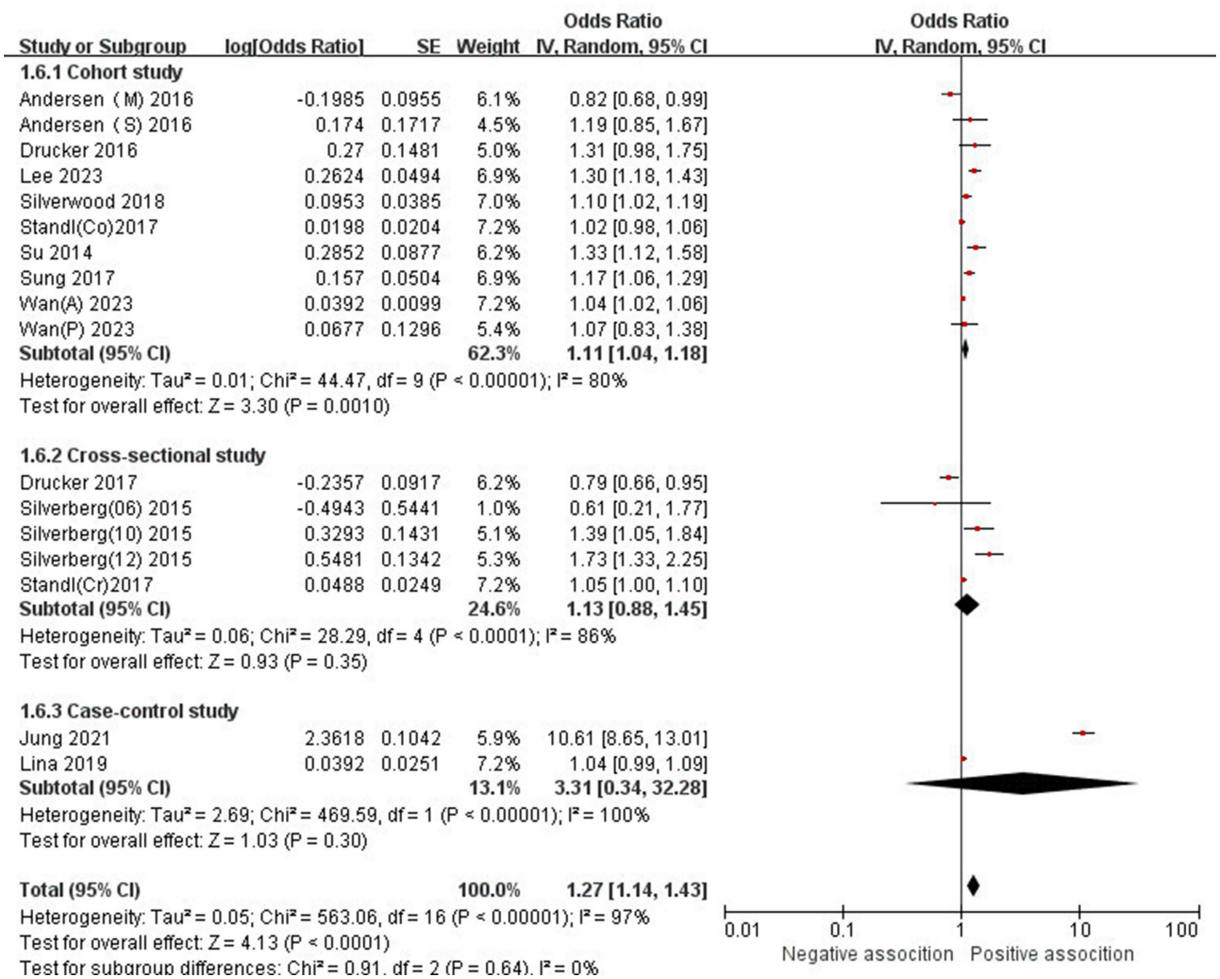
Forest plot of subgroup analysis stratified by adjustment for Study type.

**Figure 4:**
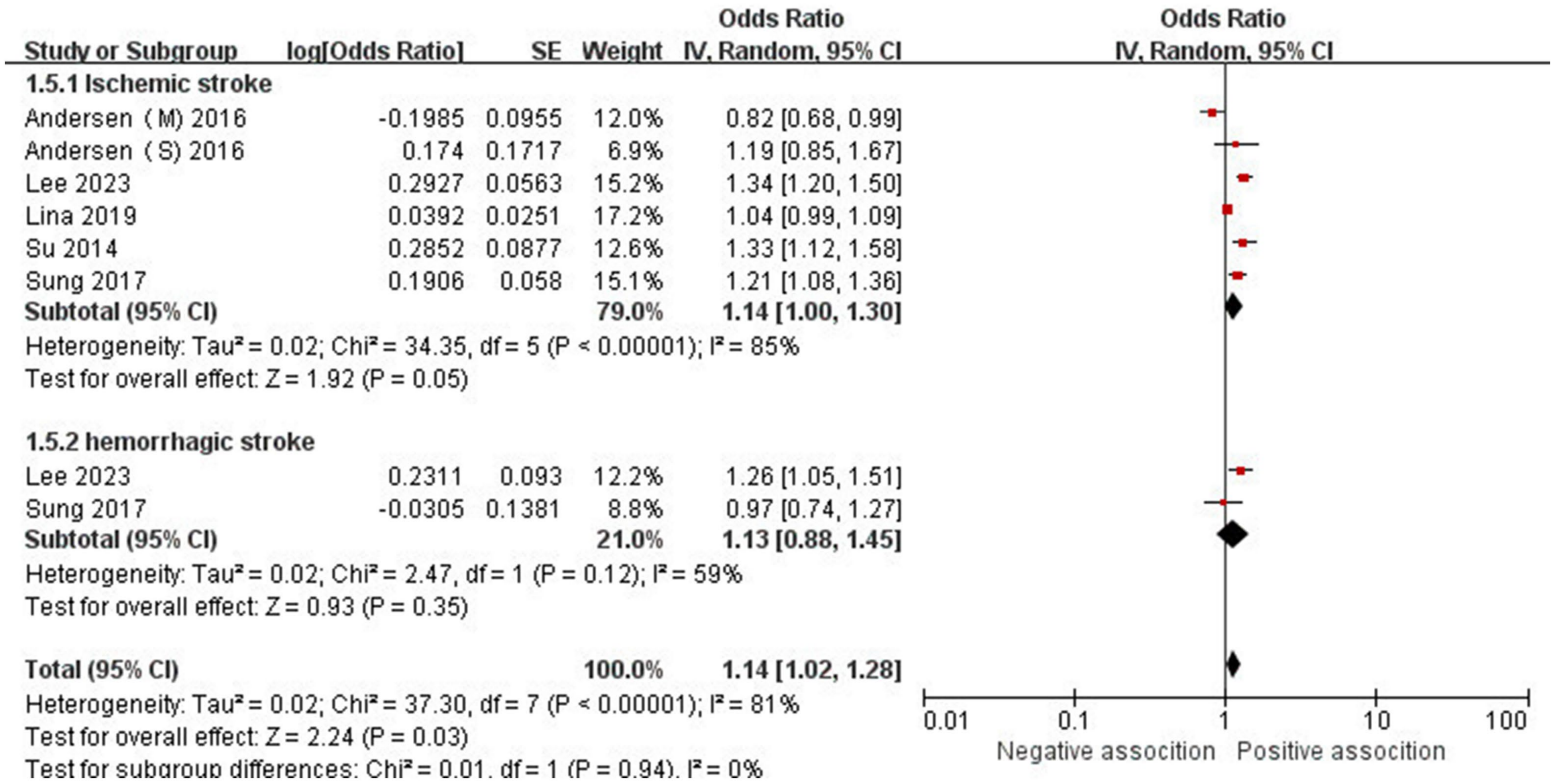
Forest plot of subgroup analysis stratified by adjustment for stroke.

**Figure 5:**
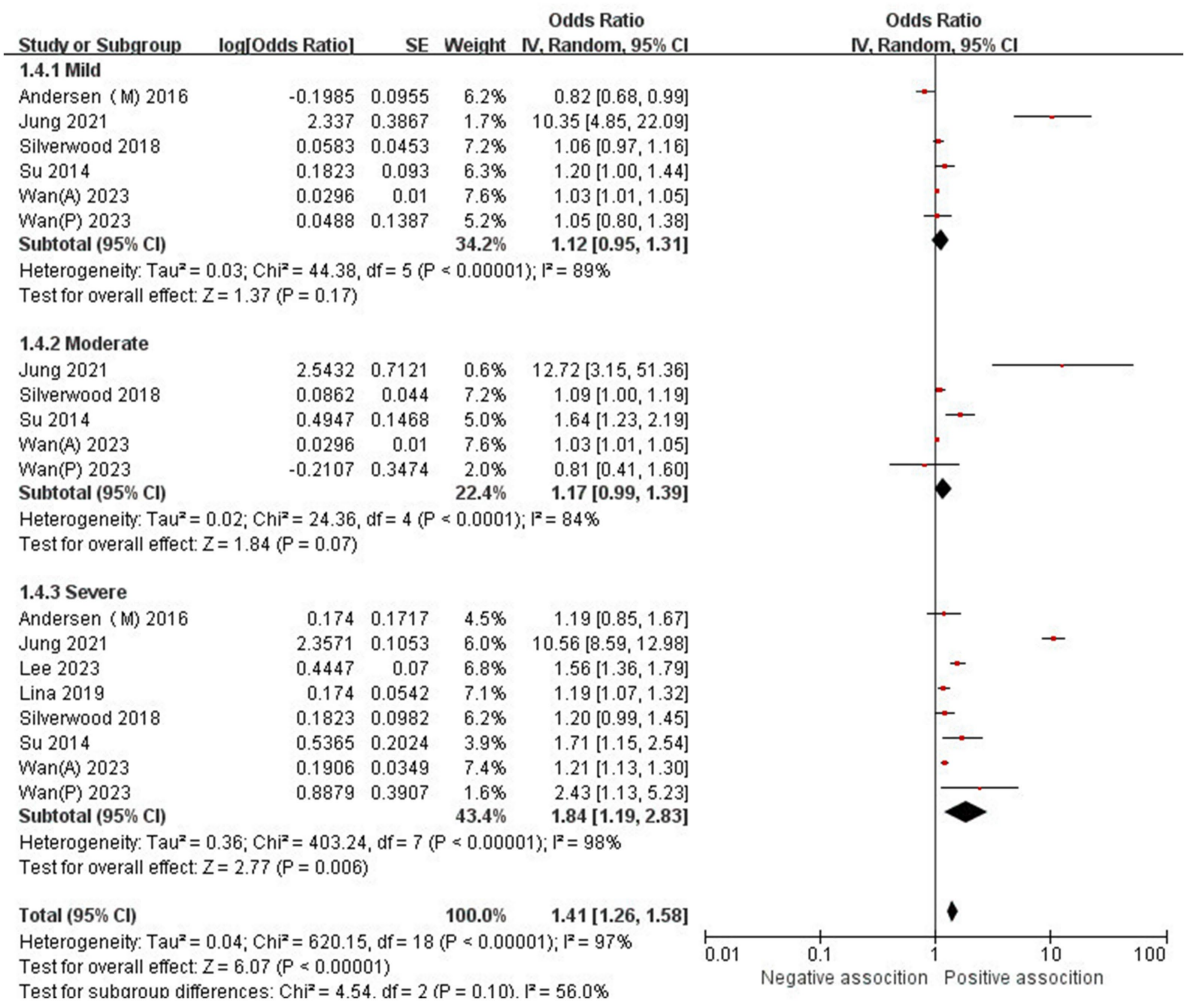
Forest plot of subgroup analysis stratified by adjustment for AD type.

**Figure 6:**
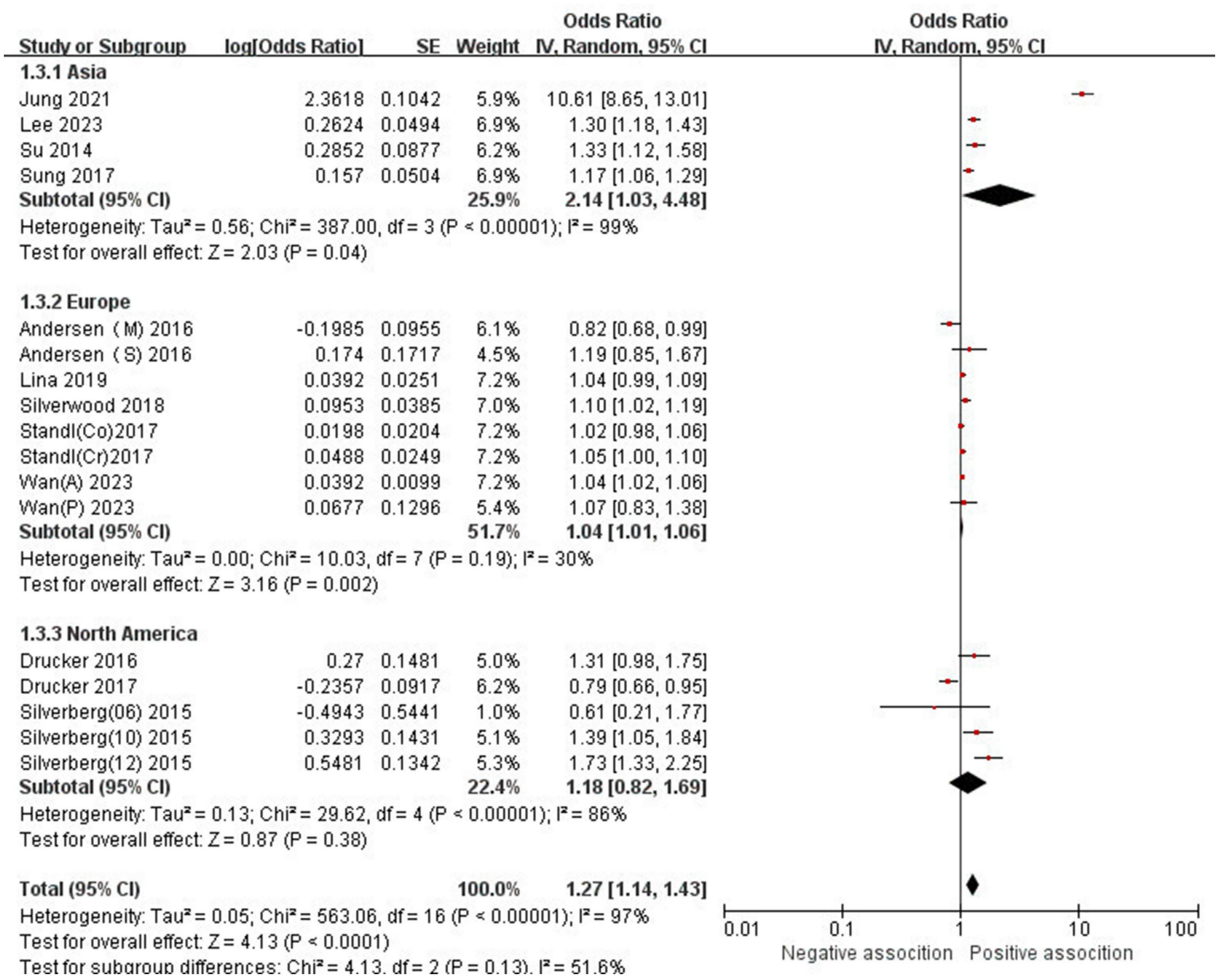
Forest plot of subgroup analysis stratified by adjustment for region.

**Figure 7:**
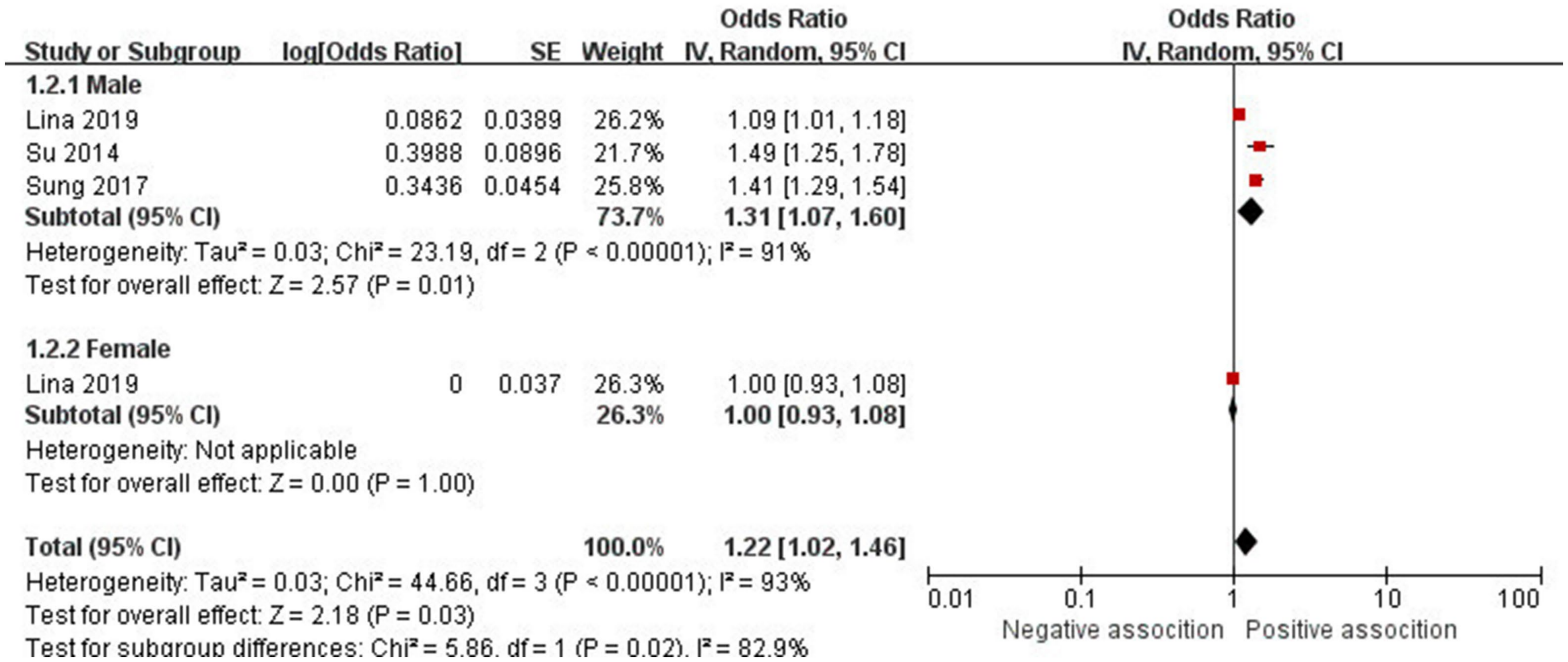
Forest plot of subgroup analysis stratified by adjustment for gender.

The association between AD and the risk of stroke has been demonstrated in 12 studies (6, 12, 13, 15–17, 24–29) comprising 17 valid datasets. A pooled analysis of these studies revealed that AD is associated with an increased risk of stroke (*n* = 17, OR = 1.27, 95%CI = 1.14–1.43) (Figure 2). However, a high degree of heterogeneity (I^2^ = 97%, *p* < 0.00001) was observed in the analysis. Due to this significant heterogeneity, we conducted a subgroup analysis to investigate its sources. Details on subgroup analyses are provided in Table 2. In our subgroup analysis categorized by study design, we identified a significant positive association between AD and the risk of stroke in eight retrospective cohort studies (12, 15, 17, 24, 26–29) (*n* = 8, OR = 1.11 95%CI = 1.04–1.18). However, no significant association was observed in the three cross-sectional studies (13, 25, 26) (*n* = 3, OR = 1.13 95%CI = 0.88–1.45) and two case–control studies (6, 16) (*n* = 2, OR = 3.31 95%CI = 0.34–32.28) (Figure 3). In the subgroup analysis stratified by stroke type, we identified a significant positive association between AD and ischemic stroke (6, 17, 24, 27, 28)(*n* = 5, OR = 1.14 95%CI = 1.00–1.30). Conversely, no significance was observed for the studies with hemorrhagic stroke (17, 28) (*n* = 2, OR = 1.13 95%CI = 0.88–1.45) (Figure 4). In the subgroup analysis stratified by type of AD, we identified a significant positive association between severe AD and stroke (6, 15–17, 24, 27, 29) (*n* = 7, OR = 1.84, 95% CI = 1.19–2.83); however, no significant association was observed with mild (15, 16, 24, 27, 29) (*n* = 5, OR = 1.12, 95% CI = 0.95–1.31) and moderate AD (15, 16, 27, 29) (*n* = 4, OR = 1.17, 95% CI = 0.99–1.39) (Figure 5). In the subgroup analysis stratified by study region, we identified a significant positive association between AD and stroke in Asia (16, 17, 27, 28) (*n* = 4, OR = 2.14, 95% CI = 1.03–4.48). However, no significant association was observed in both North America (12, 13, 25) (*n* = 3, OR = 1.18, 95% CI = 0.82–1.69) and Europe (6, 15, 24, 26, 29)(*n* = 5, OR = 1.04, 95% CI = 1.01–1.06) (Figure 6). In the subgroup analysis stratified by gender, we identified a significant positive association between AD and stroke in males (6, 27, 28)(*n* = 3, OR = 1.31, 95% CI = 1.07–1.60); however, this correlation has not been demonstrated in females (6)(*n* = 1, OR = 1.00, 95% CI = 0.93–1.08) (Figure 7).

**Table 2:** Subgroup analyses of association between AD and risk of stroke.

Subgroup	No. of studies	OR (95%CI)	Passociation	*I^2^* (%)	*P* heterogeneity
Overall studies	17	1.27 (1.14–1.43)	P<0.0001	97%	*P* < 0.00001
Gender					
Male	3	1.31 (1.07–1.60)	*p* = 0.01	91%	*P* < 0.00001
Female	1	1.00 (0.93–1.08)	*p* = 1.00	/	/
Region					
Asia	4	2.14 (1.03–4.48)	*p* = 0.04	99%	*P* < 0.00001
Europe	5	1.04 (1.01–1.06)	*p* = 0.002	30%	*p* = 0.19
North America	2	1.18 (0.82–1.69)	*p* = 0.38	86%	*P* < 0.00001
Type of AD					
Mild	5	1.12 (0.95–1.31)	*p* = 0.17	89%	*P* < 0.00001
Moderate	4	1.17 (0.99–1.39)	*p* = 0.07	84%	*p* < 0.0001
Severe	7	1.84 (1.19–2.83)	*p* = 0.006	98%	*P* < 0.00001
Type of stroke					
Ischemic stroke	5	1.14 (1.00–1.30)	*p* = 0.05	85%	*P* < 0.00001
Hemorrhagic stroke	2	1.13 (0.88–1.45)	*p* = 0.35	59%	*p* = 0.12
Type of study					
Cohort study	8	1.11 (1.04–1.18)	*p* = 0.001	80%	*P* < 0.00001
Cross-sectional study	3	1.13 (0.88–1.45)	*P* = 0.35	86%	*P* < 0.0001
Case–control study	2	3.31 (0.34–32.28)	*p* = 0.30	100%	*P* < 0.00001

HS, hemorrhagic stroke; IS, ischemic stroke; cRR: crude relative ratio; aRR, adjustment relative ratio; CI, confidence interval.

### 3.4 Sensitivity analysis

**Figure 8:**
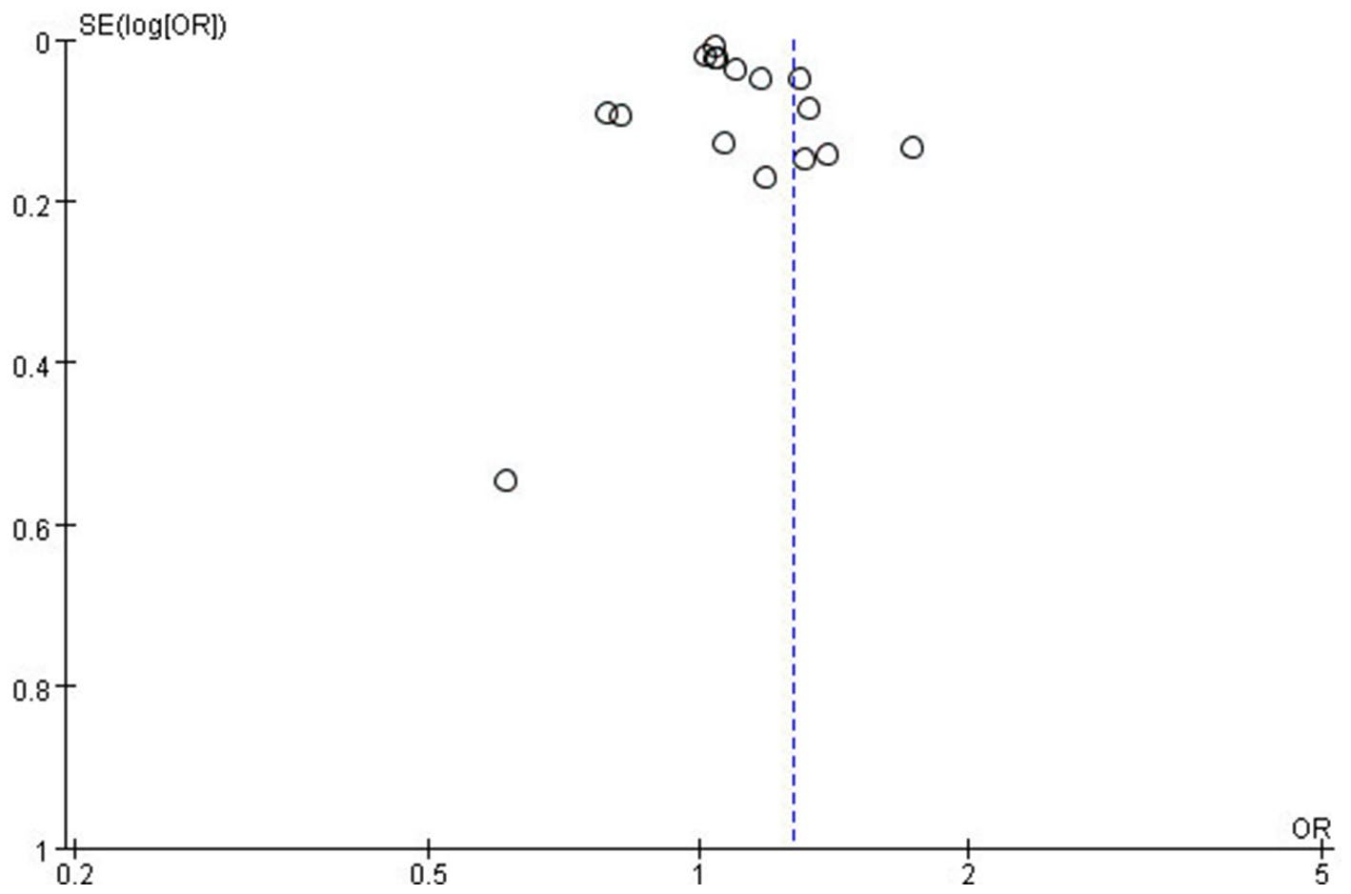
The funnel plot for publication bias.

According to the funnel plot, our team did not identify any significant publication bias (Figure 8). Additionally, we employed a one-by-one exclusion method to verify the stability of our research findings. After excluding each study individually, we observed that the results remained largely unchanged, indicating that the meta-analysis results exhibit strong stability (see Supplementary Table S5).

## 4 Discussion

### 4.1 Principal findings

Our meta-analysis primarily investigates the relationship between AD and stroke. To the best of our knowledge, this study represents the most current, extensive, and comprehensive meta-analysis investigating the relationship between AD and the risk of stroke to date. We included a total of 12 studies (6, 12, 13, 15–17, 24–29) with 14,517,146 participants, which has shown that the incidence rate of stroke in patients with AD is significantly higher than that in non-AD patients (random-effects OR = 1.27,95%CI 1.14–1.43; I^2^ = 97%). Furthermore, a sensitivity analysis was carried out by removing one study at a time, the results remained stable.

### 4.2 Comparison with previous studies

In a previous meta-analysis of observational study, Yuan et al. (14) included a total of 11 articles, comprising 11 cohort studies and 3 cross-sectional studies. The research showed that the incidence of stroke was significantly higher in patients with AD compared to non-AD patients (combined HR, 1.15; 95% CI, 1.08–1.22; *p* < 0.001). Furthermore, subgroup analyses revealed a significant correlation between severe AD and the incidence of stroke; however, this correlation was less pronounced in patients with mild or moderate AD. Additionally, AD was significantly associated with ischemic stroke, but not hemorrhagic stroke.

In comparison to previous study (14), our updated meta-analysis further substantiates these conclusions and enhances the findings of prior research.

Our study represents the most extensive investigation to date, encompassing the largest number of included studies and the most substantial sample size. Notably, it incorporates four new studies (6, 16, 17, 29) published between 2019 and 2023, thereby providing the latest and most comprehensive epidemiological evidence regarding the association between AD and stroke. Compared to Yuan’s study, we excluded Chang’s (30) study, which primarily investigated the relationship between contact dermatitis and stroke, a topic that diverges from our study’s focus. Regarding data inclusion, we integrated Andersen’s (24) findings on the relationship between non-severe AD and stroke, thereby enriching our data sources. Our literature review revealed that the data sources of Andersen’s (24) article and Egeberg (31) article may originate from the same timeframe and database; however, Andersen’s sample size for AD-related articles was significantly larger than that of Egeberg, leading to the exclusion of the latter. In summary, although our conclusions align with those of Yuan, we believe that our findings are more robust considering the aforementioned factors.

### 4.3 Potential explanations and implications

The specific pathogenesis of atopic dermatitis remains incompletely understood. It is primarily believed to involve multiple factors, including genetics, abnormal skin barrier function, microbial dysbiosis, environmental influences, and particularly immune responses (32, 33). Notably, three mechanisms of immunity are significant: (1) The Th2-type immune response predominates during acute inflammation. In this phase, Th2 cells are primarily activated, secreting cytokines such as IL-4, IL-13, and IL-31, which promote IgE production and eosinophil infiltration, resulting in itching and inflammation (34–36); (2) Th22, Th1, and Th17 cells contribute to the chronic inflammatory response. In the chronic phase, Th1 (IFN-*γ*), Th22 (IL-22), and Th17 (IL-17) cells are activated, leading to epidermal hyperplasia and sustained inflammation (37–39); (3) Other immune cells, such as Langerhans cells, mast cells, and eosinophils, also play a role in the development of atopic dermatitis (40, 41).

Current research indicates that atopic dermatitis (AD) may be implicated in the development of various diseases, including allergic asthma, inflammatory bowel disease, anxiety, and depression (42). Concurrently, the role of AD in cerebrovascular disease is garnering increasing attention. Several mechanisms have been proposed to elucidate the relationship between AD and stroke: (1) In the pathogenesis of cerebrovascular disease, inflammatory responses are critical. Studies have demonstrated that inflammatory mediators such as fractalkine/CX3CL1, CCL8, M-CSF, HGF, E-selectin, PI3/elafin, CCL17, and IL-16 are significantly elevated in patients with AD, contributing to the progression of arteriosclerosis (43–45). Additionally, PET-CT studies have revealed a significant correlation between vascular inflammation in younger AD patients and Th2-related products in both skin and blood, such as CCL17 and CCL22 (46). (2) Chronic pruritus associated with AD can lead to insomnia, anxiety, and depression in patients. This prolonged state of stress activates the sympathetic nervous system and the hypothalamic–pituitary–adrenal (HPA) axis, resulting in elevated blood pressure and vascular damage (4, 5, 7). (3) Patients with AD may exhibit obesity, hypertension, and diabetes (47, 48), all recognized risk factors for stroke. Despite numerous studies exploring the association between AD and stroke, the precise pathogenesis remains unclear, presenting a novel direction for future research.

In recent years, the correlation between AD and stroke incidence has garnered increasing attention as research in this area deepens. However, no consensus has been reached thus far. Some studies indicate a significant correlation between AD and stroke occurrence (15–17, 23), with a systematic review of 11 studies conducted by Yuan et al.in 2018 supporting this perspective (14). Conversely, the study by Drucker et al. does not corroborate this viewpoint (12, 13), and a systematic review published by Kern et al. in 2024 also fails to support this correlation (49). We included a total of 12 observational studies (6, 12, 13, 15–17, 24–29) and identified a significant correlation between AD and the occurrence of stroke. Further subgroup analysis indicated that the incidence of stroke was significantly higher in patients with severe AD compared to those with non-severe AD. This disparity may be attributed to several factors associated with severe AD, including the advanced stage of the disease, prolonged duration, obesity, anxiety, depression, insomnia, diabetes, hypertension, hyperlipidemia, and other cerebrovascular risk factors (4–7). Additionally, the inflammatory response in patients with severe AD is markedly elevated (50, 51), exacerbating the degree of atherosclerosis and consequently increasing the risk of stroke. However, due to the varying severity criteria employed across different studies, it is essential for future research to standardize these criteria to yield more credible conclusions. Compared to hemorrhagic stroke, the risk of ischemic stroke in patients with AD is significantly increased, with arteriosclerosis and thrombosis identified as the primary mechanisms underlying ischemic stroke. The systemic inflammatory response associated with AD may exacerbate this process (43, 45), thereby contributing to the elevated risk of ischemic stroke in these patients. However, due to the limited number of studies and inconsistent findings-only two articles in our research addressed hemorrhagic stroke-caution should be exercised when drawing conclusions regarding hemorrhagic stroke. Further high-quality research is essential to elucidate these relationships. Compared to females, male AD patients exhibit a stronger correlation with stroke, potentially attributable to a higher prevalence of unhealthy lifestyle habits, such as smoking, excessive alcohol consumption, late-night activities, and insufficient physical exercise. However, this conclusion remains contentious, as only one study has explored the relationship between female AD patients and stroke. Consequently, further research is warranted to elucidate this issue in future studies. In conducting subgroup analyses of research types, we found that the conclusions drawn from cohort studies regarding the significant association between AD and stroke occurrence exhibit considerably higher reliability compared to those derived from cross-sectional and case–control studies. This enhanced reliability is attributed not only to the greater number of cohort studies available but also to their superior capacity to control for confounding factors and their stronger characteristics for causal inference.

### 4.4 Strengths and limitations

This study has several strengths and limitations that warrant acknowledgment. Initially, we conducted a comprehensive literature search, applying stringent inclusion and exclusion criteria along with a rigorous quality assessment. In contrast to prior meta-analyses on this subject, our research incorporates the most recent and extensive body of literature. Furthermore, the estimated effect sizes were consistent across all included studies, thereby minimizing heterogeneity. The majority of the included studies demonstrated high quality, providing robust evidence on the topic. Lastly, due to the observed heterogeneity in results, we performed subgroup analyses based on sex, region, study type, and the classification of atopic dermatitis (AD) and stroke to explore potential sources of heterogeneity.

This meta-analysis presents several inherent limitations that warrant discussion. First, the data regarding AD, stroke, and other comorbidities were obtained from a secondary claims database. Consequently, any inaccuracies or incompleteness in these records may result in data inaccuracies. Second, while most studies extensively discuss risk factors for cerebrovascular diseases-including gender, age, hypertension, diabetes, and atrial fibrillation-this study highlights that only one article has examined the correlation between AD and female stroke. Given the limited sample size, the conclusion regarding the correlation between AD and female stroke should be interpreted with caution. It is well established that age is a significant risk factor for stroke; however, the age distribution of the population included in this meta-analysis varies, leading to ambiguous conclusions regarding the correlation between AD and stroke incidence among both elderly and middle-aged individuals. This inconsistency underscores a critical area that warrants further investigation in future research. Thirdly, our study demonstrates significant heterogeneity (I^2^ = 97%). To investigate the sources of this heterogeneity, we conducted both subgroup and sensitivity analyses. Given that all studies included in this research were observational, the heterogeneity may stem from variations in diagnostic criteria and severity grading standards for AD, differences in study regions and types, as well as adjustments for stroke types and confounding factors. Furthermore, potential confounding variables such as lack of exercise, familial genetic predisposition, hyperhomocysteinemia, staying up late, and other cerebrovascular risk factors have not been accounted for. The results may not generalize to other regions due to the limited geographic distribution of the included studies, as the majority were conducted in Asia, Europe, and North America.

## 5 Conclusion

We found a significant correlation between atopic dermatitis (AD) and the risk of stroke, particularly in the case of ischemic stroke, where this correlation is especially pronounced. However, definitive evidence regarding a correlation between AD and hemorrhagic stroke is currently lacking. To gain a deeper understanding of the relationship between AD and stroke, future prospective studies must be meticulously designed to account for confounding factors such as AD treatment status, stroke type, and geographical region.

## References

[1] Avena-Woods C. Overview of atopic dermatitis. Am J Manag Care. (2017) 23:S115–23. 28978208

[2] Cabanillas B Brehler AC Novak N. Atopic dermatitis phenotypes and the need for personalized medicine. Curr Opin Allergy Clin Immunol. (2017) 17:309–15. doi: 10.3390/jcm14093094 28582322 PMC5515628

[3] Barbarot S Auziere S Gadkari A Girolomoni G Puig L Simpson EL . Epidemiology of atopic dermatitis in adults: results from an international survey. Allergy. (2018) 73:1284–93. doi: 10.1111/all.13401, PMID: 29319189

[4] Lutgendorf SK Lamkin DM Jennings NB Arevalo JM Penedo F DeGeest K . Biobehavioral influences on matrix metalloproteinase expression in ovarian carcinoma. Clin Cancer Res. (2008) 14:6839–46. doi: 10.1158/1078-0432.CCR-08-0230, PMID: 18980978 PMC2716059

[5] Griffin GD Charron D Al-Daccak R. Post-traumatic stress disorder: revisiting adrenergics, glucocorticoids, immune system effects and homeostasis. Clin Transl Immunology. (2014) 3:e27. doi: 10.1038/cti.2014.26, PMID: 25505957 PMC4255796

[6] Ivert LU Johansson EK Dal H Lindelöf B Wahlgren CF Bradley M. Association between atopic dermatitis and cardiovascular disease: a Nationwide register-based case-control study from Sweden. Acta Derm Venereol. (2019) 99:865–70. doi: 10.2340/00015555-3235, PMID: 31197387

[7] Profumo E Maggi E Arese M Di Cristofano C Salvati B Saso L . Neuropeptide Y promotes human M2 macrophage polarization andenhances p62/SQSTM1-dependent autophagy and NRF2 activation. Int J Mol Sci. (2022) 23:13009. doi: 10.3390/ijms232113009, PMID: 36361795 PMC9653849

[8] Global, regional, and national burden of stroke and its risk factors, 1990-2019: a systematic analysis for the global burden of disease study 2019. Lancet Neurol. (2021) 20:795–820. doi: 10.1016/S1474-4422(21)00252-0, PMID: 34487721 PMC8443449

[9] Strong K Mathers C Bonita R. Preventing stroke: saving lives around the world. Lancet Neurol. (2007) 6:182–7. doi: 10.1016/S1474-4422(07)70031-5, PMID: 17239805

[10] Wu S Wu B Liu M Chen Z Wang W Anderson CS . Stroke in China: advances and challenges in epidemiology, prevention, and management. Lancet Neurol. (2019) 18:394–405. doi: 10.1016/S1474-4422(18)30500-3, PMID: 30878104

[11] Ma Q Li R Wang L Yin P Wang Y Yan C . Temporal trend and attributable risk factors of stroke burden in China, 1990-2019: an analysis for the global burden of disease study 2019. Lancet Public Health. (2021) 6:e897-e906. doi: 10.1016/S2468-2667(21)00228-0, PMID: 34838196 PMC9047702

[12] Drucker AM Li WQ Cho E Li T Sun Q Camargo CA . Atopic dermatitis is not independently associated with nonfatal myocardial infarction or stroke among US women. Allergy. (2016) 71:1496–500. doi: 10.1111/all.12957 27291834 PMC5023476

[13] Drucker AM Qureshi AA Dummer TJB Parker L Li WQ. Atopic dermatitis and risk of hypertension, type 2 diabetes, myocardial infarction and stroke in a cross-sectional analysis from the Canadian Partnership for Tomorrow Project. Br J Dermatol. (2017) 177:1043–51. doi: 10.1111/bjd.15727, PMID: 28617976

[14] Yuan M Cao WF Xie XF Zhou HY Wu XM. Relationship of atopic dermatitis with stroke and myocardial infarction: a meta-analysis. Medicine (Baltimore). (2018) 97:e13512. doi: 10.1097/MD.0000000000013512, PMID: 30544450 PMC6310567

[15] Silverwood RJ Forbes HJ Abuabara K Ascott A Schmidt M Schmidt SAJ . Severe and predominantly active atopic eczema in adulthood and long term risk of cardiovascular disease: population based cohort study. BMJ. (2018) 361:K1786. doi: 10.1136/bmj.k1786, PMID: 29792314 PMC6190010

[16] Jung HJ Lee DH Park MY Ahn J. Cardiovascular comorbidities of atopic dermatitis: using National Health Insurance data in Korea. Allergy Asthma Clin Immunol. (2021) 17:94. doi: 10.1186/s13223-021-00590-x, PMID: 34551806 PMC8456522

[17] Lee SW Kim H Byun Y Baek YS Choi CU Kim JH . Incidence of cardiovascular disease after atopic dermatitis development: a nationwide, population-based study. Allergy Asthma Immunol Res. (2023) 15:231–45. doi: 10.4168/aair.2023.15.2.231, PMID: 37021508 PMC10079521

[18] Page MJ McKenzie JE Bossuyt PM Boutron I Hoffmann TC Mulrow CD . The PRISMA 2020 statement: an updated guideline for reporting systematic reviews. BMJ. (2021) 372:n71. doi: 10.1136/bmj.n71 33782057 PMC8005924

[19] Stang A. Critical evaluation of the Newcastle-Ottawa scale for the assessment of the quality of nonrandomized studies in meta-analyses. Eur J Epidemiol. (2010) 25:603–5. doi: 10.1007/s10654-010-9491-z, PMID: 20652370

[20] Brach C Borsky A. How the U.S. Agency for Healthcare Research and Quality promotes health literate health care. Stud Health Technol Inform. (2020) 269:313–23. doi: 10.3233/SHTI200046, PMID: 32594006 PMC7413323

[21] Jing W Zhao S Liu J Liu M. ABO blood groups and hepatitis B virus infection: a systematic review and meta-analysis. BMJ Open. (2020) 10:e034114. doi: 10.1136/bmjopen-2019-034114, PMID: 32014878 PMC7045238

[22] Barrett PM McCarthy FP Kublickiene K Cormican S Judge C Evans M . Adverse pregnancy outcomes and long-term maternal kidney disease: a systematic review and meta-analysis. JAMA Netw Open. (2020) 3:e1920964. doi: 10.1001/jamanetworkopen.2019.20964, PMID: 32049292 PMC12527481

[23] Higgins JP Thompson SG Deeks JJ Altman DG. Measuring inconsistency in meta-analyses. BMJ. (2003) 327:557–60. doi: 10.1136/bmj.327.7414.557 12958120 PMC192859

[24] Andersen YMF Egeberg A Gislason GH Hansen PR Skov L Thyssen JP. Risk of myocardial infarction, ischemic stroke, and cardiovascular death in patients with atopic dermatitis. J Allergy Clin Immunol. (2016) 138:310–312.e3. doi: 10.1016/j.jaci.2016.01.015, PMID: 26971689

[25] Silverberg JI. Association between adult atopic dermatitis, cardiovascular disease, and increased heart attacks in three population-based studies. Allergy. (2015) 70:1300–8. doi: 10.1111/all.12685, PMID: 26148129

[26] Standl M Tesch F Baurecht H Rodríguez E Müller-Nurasyid M Gieger C . Association of Atopic Dermatitis with cardiovascular risk factors and diseases. J Invest Dermatol. (2017) 137:1074–81. doi: 10.1016/j.jid.2016.11.031, PMID: 28011146

[27] Su VYF Chen TJ Yeh CM Chou KT Hung MH Chu SY . Atopic dermatitis and risk of ischemic stroke: a nationwide population-based study. Ann Med. (2014) 46:84–9. doi: 10.3109/07853890.2013.870018, PMID: 24460466

[28] Sung Y-F Lin C-C Yin J-H Chou C-H Chung C-H Yang F-C . Increased risk of stroke in patients with atopic dermatitis: a population-based, longitudinal study in Taiwan. J Med Sci. (2017) 37:12–8. doi: 10.4103/1011-4564.200737, PMID: 39968893

[29] Wan J Fuxench ZCC Wang S Syed MN Shin DB Abuabara K . Incidence of cardiovascular disease and venous thromboembolism in patients with atopic dermatitis. J Allergy Clin Immunol Pract. (2023) 11:3123–3132.e3. doi: 10.1016/j.jaip.2023.08.007, PMID: 37572754

[30] Chang WL Hsu MH Lin CL Chan PC Chang KS Lee CH . Increased risk of stroke in contact dermatitis patients: a nationwide population-based retrospective cohort study. Medicine (Baltimore). (2017) 96:e5650. doi: 10.1097/MD.0000000000005650, PMID: 28272195 PMC5348143

[31] Egeberg A Andersen YM Gislason GH Skov L Thyssen JP. Prevalence of comorbidity and associated risk factors in adults with atopic dermatitis. Allergy. (2017) 72:783–91. doi: 10.1111/all.13085, PMID: 27864954

[32] Goleva E Berdyshev E Leung DY. Epithelial barrier repair and prevention of allergy. J Clin Invest. (2019) 129:1463–74. doi: 10.1172/JCI124608, PMID: 30776025 PMC6436854

[33] Kim J Kim BE Leung DYM. Pathophysiology of atopic dermatitis: clinical implications. Allergy Asthma Proc. (2019) 40:84–92. doi: 10.2500/aap.2019.40.4202, PMID: 30819278 PMC6399565

[34] Paul WE Zhu J. How are T(H)2-type immune responses initiated and amplified? Nat Rev Immunol. (2010) 10:225–35. doi: 10.1038/nri2735, PMID: 20336151 PMC3496776

[35] Kaplan MH. Th9 cells: differentiation and disease. Immunol Rev. (2013) 252:104–15. doi: 10.1111/imr.12028, PMID: 23405898 PMC3982928

[36] Licona-Limón P Kim LK Palm NW Flavell RA. TH2, allergy and group 2 innate lymphoid cells. Nat Immunol. (2013) 14:536–42. doi: 10.1038/ni.2617, PMID: 23685824

[37] Res PC Piskin G de Boer OJ van der Loos CM Teeling P Bos JD . Overrepresentation of IL-17A and IL-22 producing CD8 T cells in lesional skin suggests their involvement in the pathogenesis of psoriasis. PLoS One. (2010) 5:e14108. doi: 10.1371/journal.pone.0014108 21124836 PMC2991333

[38] Szegedi K Lutter R Res PC Bos JD Luiten RM Kezic S . Cytokine profiles in interstitial fluid from chronic atopic dermatitis skin. J Eur Acad Dermatol Venereol. (2015) 29:2136–44. doi: 10.1111/jdv.13160, PMID: 25980674

[39] Esaki H Brunner PM Renert-Yuval Y Czarnowicki T Huynh T Tran G . Early-onset pediatric atopic dermatitis is T(H)2 but also T(H)17 polarized in skin. J Allergy Clin Immunol. (2016) 138:1639–51. doi: 10.1016/j.jaci.2016.07.013, PMID: 27671162

[40] Brandt EB Sivaprasad U. Th2 cytokines and atopic dermatitis. J Clin Cell Immunol. (2011) 2:110. doi: 10.4172/2155-9899.1000110 PMC3189506 21994899

[41] Beyer L Kabatas AS Mommert S Stark H Werfel T Gutzmer R . Histamine activates human eosinophils via H(2)R and H(4)R predominantly in atopic dermatitis patients. Int J Mol Sci. (2022) 23:10924. doi: 10.3390/ijms231810294, PMID: 36142206 PMC9499661

[42] Narla S Silverberg JI. Association between atopic dermatitis and autoimmune disorders in US adults and children: a cross-sectional study. J Am Acad Dermatol. (2019) 80:382–9. doi: 10.1016/j.jaad.2018.09.025, PMID: 30287311

[43] Shah R Matthews GJ Shah RY McLaughlin C Chen J Wolman M . Serum Fractalkine (CX3CL1) and cardiovascular outcomes and diabetes: findings from the chronic renal insufficiency cohort (CRIC) study. Am J Kidney Dis. (2015) 66:266–73. doi: 10.1053/j.ajkd.2015.01.021, PMID: 25795074 PMC4516570

[44] Brunner PM Suárez-Fariñas M He H Malik K Wen HC Gonzalez J . The atopic dermatitis blood signature is characterized by increases in inflammatory and cardiovascular risk proteins. Sci Rep. (2017) 7:8707. doi: 10.1038/s41598-017-09207-z, PMID: 28821884 PMC5562859

[45] Kassam HA Gillis DC Dandurand BR Karver MR Tsihlis ND Stupp SI . Development of fractalkine-targeted nanofibers that localize to sites of arterial injury. Nanomaterials. (2020) 10:420. doi: 10.3390/nano10030420, PMID: 32121105 PMC7152859

[46] Villani AP Pavel AB Wu J Fernandes M Maari C Saint-Cyr Proulx E . Vascular inflammation in moderate-to-severe atopic dermatitis is associated with enhanced Th2 response. Allergy. (2021) 76:3107–21. doi: 10.1111/all.14859 33866573

[47] Eller E Kjaer HF Høst A Andersen KE Bindslev-Jensen C. Development of atopic dermatitis in the DARC birth cohort. Pediatr Allergy Immunol. (2010) 21:307–14. doi: 10.1111/j.1399-3038.2009.00914.x, PMID: 19788539

[48] Kwa MC Silverberg JI. Association between inflammatory skin disease and cardiovascular and cerebrovascular co-morbidities in US adults: analysis of Nationwide inpatient sample data. Am J Clin Dermatol. (2017) 18:813–23. doi: 10.1007/s40257-017-0293-x, PMID: 28534318

[49] Kern C Ortiz C Johanis M Ye M Tahir P Mulick A . Atopic dermatitis and cardiovascular risk in pediatric patients: a systematic review and meta-analysis. J Invest Dermatol. (2024) 144:e1016:1038–47. doi: 10.1016/j.jid.2023.09.285 PMC11163969 37972725

[50] Steyers CM 3rd Miller FJ Jr. Endothelial dysfunction in chronic inflammatory diseases. Int J Mol Sci. (2014) 15:11324–49. doi: 10.3390/ijms150711324, PMID: 24968272 PMC4139785

[51] Weidinger S Beck LA Bieber T Kabashima K Irvine AD. Atopic dermatitis. Nat Rev Dis Primers. (2018) 4:1. doi: 10.1038/s41572-018-0001-z, PMID: 29930242

