# Supplementary material for: Association between atopic dermatitis and risk of stroke: a systematic review and meta-analysis
Source: Front Neurol. 2025 Jul 18;16:1630671. doi: 10.3389/fneur.2025.1630671 (PMC12316183; doi:10.3389/fneur.2025.1630671)
Supplement: Supplementary file 1 [file Supplementary_file_1.pdf]

Supplementary online material		
**Content**		**Page**

**Table S1.**	Representatives search strings for PubMed	2
**Table S2.**	Description of excluded studies	3-4
**Table S3.**	The other Characteristics of included studies	5-7
**Table S4.**	Methodological quality assessment of included studies with NOS and AHRQ	8
**Table S5.**	Results of sensitivity analyses	9

Table S1. Representatives search strings for PubMed (From inception to February 2025)	
**Databases**	**Search strings**
**PubMed**	(((((((("Stroke"[Mesh]) OR (cerebral infarction[Title/Abstract])) OR (brain infarction[Title/Abstract])) OR (cerebral hemorrhage[Title/Abstract])) OR (intracerebral hemorrhage[Title/Abstract])) OR (transient ischemic attack[Title/Abstract])) OR (cerebrovascular disorders[Title/Abstract])) OR (cerebrovascular accident[Title/Abstract])) AND (((("Dermatitis, Atopic"[Mesh]) OR (Dermatitis[Title/Abstract])) OR (Atopic Eczema[Title/Abstract])) OR (Eczema[Title/Abstract]))

**Table S2. Description of excluded studies at the stage of eligibility according to the PRISMA flow chart.**			
**No.**	**First author**	**Publication year**	**Reason for exclusion**
1.	Chen	2009	Patients without AD
2.	Schmitt	2010	Irrelevant studies
3.	Chiu	2018	Irrelevant studies
4.	Brunner	2017	Review
5.	Davis	2022	Review
6.	Abedi	2021	Review
7.	Tan	2021	Review
8.	Davis	2022	Review
9	Qi	2022	Review
10	Akins	2024	Case report
11	Ingram	2018	Patients without AD
12	Kern	2024	Review
13	Setyawan	2021	Irrelevant studies
14	Ankur	2022	Irrelevant studies

References for the table S2

1.Chen YH, Lee HC, Lin HC. Prevalence and risk of atopic disorders among schizophrenia patients: a nationwide populatio based study. Schizophr Res. 2009 Mar;108(1-3):191-6. doi: 10.1016/j.schres.2008.12.021. Epub 2009 Jan 25. PMID: 19171465.

2.Schmitt J, Ford DE. Psoriasis is independently associated with psychiatric morbidity and adverse cardiovascular risk factors, but not with cardiovascular events in a population-based sample. J Eur Acad Dermatol Venereol. 2010 Aug;24(8):885-92. doi: 10.1111/j.1468-3083.2009.03537.x. Epub 2009 Dec 10. PMID: 20015170.

3.Chiu HY, Muo CH, Sung FC. Associations of chronic urticaria with atopic and autoimmune comorbidities: a nationwide population-based study. Int J Dermatol. 2018 Jul;57(7):822-829. doi: 10.1111/ijd.14000. Epub 2018 Apr 16. PMID: 29663342.

4.Brunner PM, Suárez-Fariñas M, He H, Malik K, Wen HC, Gonzalez J, Chan TC, Estrada Y, Zheng X, Khattri S, Dattola A, Krueger JG, Guttman-Yassky E. The atopic dermatitis blood signature is characterized by increases in inflammatory and cardiovascular risk proteins. Sci Rep. 2017 Aug 18;7(1):8707. doi: 10.1038/s41598-017-09207-z. Erratum in: Sci Rep. 2018 May 29;8(1):8439. doi: 10.1038/s41598-018-26378-5. PMID: 28821884; PMCID: PMC5562859.

5.Davis DMR, Drucker AM, Alikhan A, Bercovitch L, Cohen DE, Darr JM, Eichenfield LF, Frazer-Green L, Paller AS, Silverberg JI, Singh AM, Sidbury R. American Academy of Dermatology Guidelines: Awareness of comorbidities associated with atopic dermatitis in adults. J Am Acad Dermatol. 2022 Jun;86(6):1335-1336.e18. doi: 10.1016/j.jaad.2022.01.009. Epub 2022 Jan 24. PMID: 35085682.

6.Lu J, Ma Y, Wu J, Huang H, Wang X, Chen Z, Chen J, He H, Huang C. A review for the neuroprotective effects of andrographolide in the central nervous system. Biomed Pharmacother. 2019 Sep;117:109078. doi: 10.1016/j.biopha.2019.109078. Epub 2019 Jun 7. PMID: 31181444.

7. Tan L, Song X, Ren Y, Wang M, Guo C, Guo D, Gu Y, Li Y, Cao Z, Deng Y. Anti-inflammatory effects of cordycepin: A review. Phytother Res. 2020 Oct 8. doi: 10.1002/ptr.6890. Epub ahead of print. PMID: 33090621.

8. Davis DMR, Drucker AM, Alikhan A, Bercovitch L, Cohen DE, Darr JM, Eichenfield LF, Frazer-Green L, Paller AS, Silverberg JI, Singh AM, Sidbury R. American Academy of Dermatology Guidelines: Awareness of comorbidities associated with atopic dermatitis in adults. J Am Acad Dermatol. 2022 Jun;86(6):1335-1336.e18. doi: 10.1016/j.jaad.2022.01.009. Epub 2022 Jan 24. PMID: 35085682.

9.Qi H, Wang L, Li L. Association Between Atopic Dermatitis and Major Cardiovascular Outcomes: a Two-Sample Mendelian Randomization Study. Dermatol Pract Concept. 2022 Oct 1;12(4):e2022165. doi: 10.5826/dpc.1204a165. PMID: 36534570; PMCID: PMC9681179.

10.Akins PT. The sentinel rash and neurocutaneous presentation of atrial myxoma: Case report and literature review. Heliyon. 2024 Feb 1;10(4):e25387. doi: 10.1016/j.heliyon.2024.e25387. PMID: 38375268; PMCID: PMC10875371.

11. Ingram JR. Atopic eczema and cardiovascular disease. BMJ. 2018 May 23;361:k2064. doi: 10.1136/bmj.k2064. PMID: 29794003.

12.Kern C, Ortiz C, Johanis M, Ye M, Tahir P, Mulick A, Allen IE, McCulloch CE, Langan SM, Abuabara K. Atopic Dermatitis and Cardiovascular Risk in Pediatric Patients: A Systematic Review and Meta-Analysis. J Invest Dermatol. 2024 May;144(5):1038-1047.e16. doi: 10.1016/j.jid.2023.09.285. Epub 2023 Nov 14. PMID: 37972725; PMCID: PMC11163969.

13.Setyawan J, Mu F, Yarur A, Zichlin ML, Yang H, Fernan C, Billmyer E, Downes N, Azimi N, Strand V. Risk of Thromboembolic Events and Associated Risk Factors, Including Treatments, in Patients with Immune-mediated Diseases. Clin Ther. 2021 Aug;43(8):1392-1407.e1. doi: 10.1016/j.clinthera.2021.06.008. Epub 2021 Jul 6. PMID: 34238587.

14. Jindal AK, Rastogi P, Anjani G, Rikhi R, Rawat A, Ahluwalia J. An Autopsy Case of Wiskott-Aldrich Syndrome Revealing "FDC-Only Lymphoid Follicles" in Lymphoid Tissue: A Morphologic Correlate of Defective Immune Synapse. Pediatr Dev Pathol. 2022 May-Jun;25(3):345-350. doi: 10.1177/10935266211058345. Epub 2022 Mar 2. PMID: 35236172.

Table S3 The Characteristics of included studies about continents, study subjects, confounders adjustment and corresponding data in the meta-analysis				
**First author (year)**	**Continents**	**Study subjects**	**Confounders adjustment**	**OR/HR (95%CI)**
Andersen 2016	Europe	DNR	Age, sex, socioeconomic status, smoking, comorbidities, and medication use	AD (mild): IRR:0.82(0.68-0.98) AD (severe): IRR:1.19(0.85-1.65)
Drucker 2016	North America	NHS2	Age, race, BMI, physical activity, alcohol intake, smoking status, hormone replacement use, family history of MI, hypertension, hypercholesterolemia, diabetes	OR:1.31 (0.98-1.76)
Drucker 2017	North America	CPTP	Age, sex, hypertension, type2 diabetes, myocardial infarction, ethnic background, BMI, smoking 100cigarettes, weekly alcohol intake, average daily sleep, weekly physical activity, history of asthma	OR:0.79 (0.66-0.95)
Jung 2021	Asia	KNHIRD	Sex, age, and other CVD and metabolic diseases	HR:10.61 (8.65-13.03)
Lee 2023	Asia	NHISNSC	Sex, age, household income, region of residence, body mass index, smoking status, comorbidities (hypertension, diabetes, and hyperlipidemia), Charlson comorbidity index, and co-medications	HR:1.30 (1.18-1.44) HR (IS):1.34 (1.20-1.49) HR(HS):1.26(1.05-1.52)

Lina 2019	Europe	SNRD	Diabetes mellitus, hyperlipidemia, hypertension and years of education	OR:1.04(0.99-1.09)
Silverberg 2015	North America	NHIS, NHANES	Age, sex, race, level of education, household income,1-yearhistoryof asthma and hay fever,BMI, smoking, alcohol consumption, frequency of vigorous physical activity	OR (06): 0.61(0.21–1.80)<br>OR (10): 1.39(1.05–1.83)<br>OR (12): 1.73(1.33–2.25)
Silverwood 2018	Europe	CPRD	Body mass index and smoking at cohort entry, and time-varying hyperlipidaemia, hypertension, depression, anxiety, diabetes, and several alcohol use	aOR:1.10 (1.02-1.19)
Standl 2017	Europe	AOKPLUS	Sex, cubic age, and socioeconomic status of region and access to healthcare	RR:(Cr)1.05(1.00-1.11)<br>RR(Co):1.02(0.98-1.07)
Su 2014	Asia	NHRI	Age, sex, comorbidities, and medications and competing risk	HR:1.33(1.12-1.59)<br>AD (mild)HR:1.20(1.00-1.45)<br>AD (moderate)HR:1.33(1.12-1.59)<br>AD (severe)HR:1.33(1.12-1.59)
Sung 2017	Asia	LHID	Age, sex, hypertension, diabetes mellitus, dyslipidemia, coronary artery disease, atrial fibrillation	HR:1.17(1.06-1.30)<br>AD (IS)HR:1.21(1.08-1.36)<br>AD (HS)HR:0.97(0.74-1.29)
Wan 2023	Europe	THIN	Age, sex, Townsend score, body mass index, smoking and alcohol status	Pediatric cohorts:<br>AD (all): HR:1.07(0.83-1.36)<br>AD (mild)HR:1.05 (0.80-1.37)<br>AD (moderate)HR:0.81(0.41-1.57)<br>AD (severe)HR:2.43 (1.13-5.22)<br>Adult cohorts:<br>AD (all): HR:1.04(1.02-1.06)<br>AD (mild)HR:1.03 (1.01-1.06)<br>AD (moderate)HR:1.03 (1.01-1.06)<br>AD (severe)HR:1.21 (1.13-1.30)

CPTP: Canadian Partnership for Tomorrow Project, CPRD: Clinical Practice Research Datalink, DNR: Danish Nationwide Registers, KNHIRD: Korean National Health Insurance Research Database, LHID: Longitudinal Health InsuranceDatabase,NHS2: The Nurses' Health Study2, NHISNSC: National Health Insurance Service-National Sample Cohort, NHIS: National Health Interview Survey, NHANES: National Health and Nutrition Examination Survey, NHRI: National Health Research Institute, SNRD: Swedish National Register Data, THIN :The Health Improvement Network

Table S4 Methodological quality assessment of included studies with NOS.

First, author	Selection	Comparability	Exposure/Outcome	Total (0-9)	Quality
Andersen 2016	❉❉❉	❉❉	❉❉❉	8	High
Drucker 2016	❉❉❉❉	❉❉	❉❉❉	9	High
Jung 2021	❉❉❉	❉❉	❉❉❉	8	High
Lee 2023	❉❉❉❉	❉❉	❉❉❉	9	High
Lina 2019	❉❉❉	❉❉	❉❉❉	8	High
Silverwood 2018	❉❉❉❉	❉❉	❉❉❉	9	High
Standl 2017(Co)	❉❉❉	❉❉	❉❉	7	High
Su 2014	❉❉❉	❉❉	❉❉❉	8	High
Sung 2017	❉❉❉	❉❉	❉❉❉	8	High
Wan 2023	❉❉❉	❉❉	❉❉	7	High

Methodological quality assessment of included studies with AHRQ

First, author	1	2	3	4	5	6	7	8	9	10	11	Total (0-11)	Quality
Drucker 2017	Y	Y	U	Y	N	Y	Y	Y	N	N	Y	7	moderate
Silverberg 2015	Y	Y	Y	U	N	Y	N	Y	U	N	Y	6	moderate
Standl 2017(Cr)	Y	Y	Y	Y	N	Y	U	Y	U	U	Y	7	moderate

Note: NOS, Newcastle–Ottawa Scale. AHRQ: Agency for Healthcare Research and Quality
Y: yes, N:no, U: unclear 1-11: the item number in AHRQ

Table S5 Results of sensitivity analyses			
**Studies omitted**	**OR (95% CI)**	$P_{association}$	**Heterogeneity**
Andersen(M)2016	1.31(1.16-1.48)	P < 0.00001	*97%*
Andersen (S) 2016	1.28(1.14-1.44)	P < 0.0001	*97%*
Drucker 2016	1.27(1.13-1.43)	P < 0.0001	*97%*
Drucker 2017	1.31(1.17-1.48)	P < 0.00001	*97%*
Jung 2021	1.09(1.04-1.15)	P=0.0004	*79%*
Lee 2023	1.27(1.13-1.43)	P < 0.0001	*97%*
Lina 2019	1.30(1.14-1.48)	P < 0.0001	*97%*
Silverberg (06) 2015	1.28(1.14-1.44)	P < 0.0001	*97%*
Silverberg (10) 2015	1.27(1.13-1.43)	P < 0.0001	*97%*
Silverberg (12) 2015	1.25(1.11-1.41)	P=0.0002	*97%*
Silverwood 2018	1.29(1.14-1.46)	P < 0.0001	*97%*
Standl (Co)2017	1.30(1.13-1.49)	P=0.0002	*97%*
Standl (Cr)2017	1.29(1.14-1.47)	P < 0.0001	*97%*
Su 2014	1.27(1.13-1.43)	P < 0.0001	*97%*
Sung 2017	1.28(1.14-1.45)	P < 0.0001	*97%*
Wan(A) 2023	1.30(1.11-1.51)	P=0.001	*97%*
Wan(P) 2023	1.29(1.14-1.45)	P < 0.0001	*97%*
